# Expression of adiponectin receptors in human and rat intervertebral disc cells and changes in receptor expression during disc degeneration using a rat tail temporary static compression model

**DOI:** 10.1186/s13018-016-0481-z

**Published:** 2016-11-22

**Authors:** Yoshiki Terashima, Kenichiro Kakutani, Takashi Yurube, Toru Takada, Koichiro Maeno, Hiroaki Hirata, Shingo Miyazaki, Masaaki Ito, Yuji Kakiuchi, Yoshiki Takeoka, Ryosuke Kuroda, Kotaro Nishida

**Affiliations:** Department of Orthopaedic Surgery, Kobe University Graduate School of Medicine, 7-5-1 Kusunoki-cho, Chuo-ku, Kobe, 650-0017 Japan

**Keywords:** Adiponectin, Adiponectin receptor, Anti-inflammatory effect, Intervertebral disc, Degeneration, Animal model

## Abstract

**Background:**

Adipose tissue is a large endocrine organ known to secret adiponectin, which has anti-diabetic, anti-atherogenic, and anti-inflammatory properties. Adiponectin is widely involved in systemic disease, diabetes mellitus, and cardiac infraction. This study aimed to investigate the involvement of adiponectin in intervertebral disc (IVD) degeneration.

**Methods:**

Adipose and IVD tissues were obtained from human patients undergoing surgery (*n* = 4) and from skeletally mature Sprague–Dawley rats (*n* = 21). Tissues were stained immunohistochemically for adiponectin and adiponectin receptors AdipoR1 and AdipoR2. Changes in adiponectin receptor expression with IVD degeneration severity were then investigated using a rat tail temporary compression model. Rat IVD tissues were stained immunohistochemically with AdipoR1 or AdipoR2, and immunopositive cell percentages were calculated. Rat nucleus pulposus (NP) and annulus fibrosus (AF) tissues were isolated separately and treated with recombinant adiponectin (Ad 0.1 or 1.0 μg/ml) and/or interleukin-1 beta (IL-1β) (0.2 μg/ml) for 24 h. The four groups were as follows: control group (no treatment), IL-1β group (IL-1β-only treatment), IL-1β+Ad (0.1) group (IL-1β and adiponectin [0.1 μg/ml] treatment), and IL-1β+Ad (1.0) group (IL-1β and adiponectin [1.0 μg/ml]). Real-time reverse transcription-polymerase chain reaction was performed to evaluate messenger-RNA (mRNA) expression of tumor necrosis factor-alpha (TNF-α) and interleukin-6 (IL-6).

**Results:**

Adiponectin was widely expressed in human subcutaneous and epidural adipose tissue. In rat IVD tissue, adiponectin was not observed in NP and AF. However, both AdipoR1 and AdipoR2 were widely expressed in both human and rat IVD tissues, with no significant differences in expression levels between receptors. Furthermore, expression levels of AdipoR1 and AdipoR2 were gradually decreased with increased IVD degeneration severity. Interestingly, mRNA expression levels of TNF-α and IL-6 were significantly upregulated by IL-1β stimulation. TNF-α expression in the IL-1β+Ad 1.0 group was significantly lower than that in the IL-1β group in both NP and AF cells (*P* < 0.05). Finally, IL-6 expression was not affected by adiponectin treatment in IVD cells.

**Conclusions:**

This study investigated for the first time the expression of adiponectin receptors in human and rat IVD cells. The findings indicate that adiponectin produced by the systemic or epidural adipose tissue may be involved in the pathomechanism of IVD degeneration.

## Background

Low back pain (LBP) is a highly debilitating disorder that significantly impacts the workforce and increases medical expenditure, resulting in high socioeconomic costs worldwide [1]. Intervertebral disc (IVD) degeneration is considered to be a major cause of LBP [2]. The cause of IVD degeneration is multifactorial and heritable, with overweight or obese conditions as known risk factors [3]. Diabetes mellitus (DM) is also a global health problem, with several investigators recently reporting the involvement of systemic diseases, like DM, in LBP [4, 5]. However, there are few reports concerning the relationship between DM and IVD degeneration [6, 7]; therefore, this research topic remains unclear.

Adipose tissue is a large endocrine organ that secrets various bioactive molecules, known as adipokines, including tumor necrosis factor-α (TNF-α), adipsin, leptin, resistin, and adiponectin [8]. Among these adipokines, adiponectin was originally identified independently by four researchers using different approaches [9, 10] and is known to have anti-diabetic, anti-atherogenic, and anti-inflammatory effects [11, 12]. Adiponectin exists in abundance in the blood circulation. Interestingly, it is reported that the plasma adiponectin level is remarkably reduced in patients with DM [8].

Two subtypes of adiponectin receptor (AdipoR1 and AdipoR2) have been identified by expression cloning of complementary DNA encoding [13]. Both AdipoR1 and AdipoR2 contain seven-transmembrane domains but are structurally and functionally distinct from G protein-coupled receptors [13]. AdipoR1 and AdipoR2 serve as the major receptors for adiponectin in vivo, with AdipoR1 activating activated protein kinase (AMPK) pathways and AdipoR2 activating peroxisome proliferator-activated receptor alpha (PPAR-α) pathways [14]. Recently, a number of studies have reported adiponectin receptor expression in various organs such as the brain [15], adipose tissue [16], and cartilage [17]. However, expression of adiponectin in the IVD tissue of humans or rats has not been previously investigated.

Patients with DM, presenting low-level plasma adiponectin, often present with LBP [4, 5]. In the current study, we hypothesized that the adiponectin pathway, and associated anti-inflammatory effect, may be associated with IVD degeneration. We first assessed expression levels of AdipoR1 and AdipoR2 in both human and rat IVD cells. To determine the involvement of adiponectin in IVD degeneration, we assessed changes in receptor expression with disc degeneration using a rat tail temporary static compression model, which reproduces different stages of IVD degeneration [18]. The anti-inflammatory property of adiponectin on IVD cells was also investigated.

## Methods

### Ethics statement

The collection and use of human IVD specimens were approved by the Ethics Committee of Kobe University Hospital. All samples were obtained in accordance with the World Medical Association Declaration of Helsinki Ethical Principles for Medical Research Involving Human Subjects. This study was approved by the Institutional Review Board of Kobe University Graduate School of Medicine. All patients provided written informed consent. All animal procedures were performed under the approval and guidance of the Animal Care and Use Committee at the authors’ institution. Skeletally mature male Sprague–Dawley (SD) rats (12 weeks old; CLEA Japan, Tokyo, Japan) were used.

### Assessment of adiponectin expression in human adipose and rat IVD tissues

Subcutaneous and epidural adipose tissues were obtained from four consenting patients (two males and two females, 65.0 ± 12.4 years, range 48–77 years) during surgical procedure (lumbar disc herniation: two cases; lumbar spinal stenosis: two cases). None of the patients had systemic diseases such as DM or rheumatoid arthritis. Samples were fixed in 4% paraformaldehyde (PFA) and rapidly frozen for ~15 s in liquid nitrogen prior to being sectioned at a thickness of 15 μm.

IVD tissue was obtained from four male SD rats. IVD tissue samples were fixed in 4% PFA and decalcified in 10% ethylenediaminetetraacetic acid (EDTA). After being dehydrated in a graded series of alcohol, they were embedded in paraffin and sectioned at 6-μm thickness. Samples were then deparaffinized in xylene and rehydrated in a graded series of alcohol. All sections were stained overnight (4 °C) with anti-mouse adiponectin (Abcam, MA, USA) at a dilution of 1:100. After primary antibody incubation, samples were washed three times with phosphate-buffered saline (PBS) and incubated for 1 h with secondary antibody (Molecular Probes, OR, USA).

### Immunohistochemical assessment of AdipoR1 and AdipoR2 expression in human and rat IVD tissues

Four human IVD tissue samples were obtained from the same four patients and during the same surgical procedures as detailed above. All IVD tissue samples were fixed in 4% PFA and decalcified in 10% EDTA. After being dehydrated in a graded series of alcohol, the samples were embedded in paraffin and sectioned at 6-μm thickness. The samples were then deparaffinized in xylene and rehydrated in a graded series of alcohol. The sections were divided into two groups: one group was stained with anti-goat AdipoR1 (Santa Cruz, CA, USA) and the other with anti-rabbit AdipoR2 (Santa Cruz, CA, USA) antibodies. These sections were incubated with the primary antibodies at a dilution of 1:100 at 4 °C overnight. Subsequently, the sections were treated at room temperature for 1 h with peroxidase-labeled anti-goat or anti-rabbit antibodies (Nichirei Bioscience, Tokyo, Japan), respectively. A brown reaction product was developed using peroxidase substrate 3,3′-diaminobenzidine (DAKO), and counterstaining was performed with hematoxylin. Images were immediately captured using a BZ-9000 microscope (Keyence). Specimens were evaluated and scored based on a semiquantitative approach to determine the percentage (%) of AdipoR1- and AdipoR2-positive cells. Numbers of immunostained cells were counted in at least three randomly selected high-power fields of view (150 cells or more/view) for each sample.

SD rat coccygeal IVD tissues (*n* = 4 animals; vertebral body–disc–vertebral body) were obtained en bloc and immunostained with AdipoR1 and AdipoR2 using the aforementioned procedures.

### Changes in adiponectin receptor expression using a rat tail temporary static compression model

SD rats (*n* = 9) underwent intraperitoneal anesthesia, and radiographs were taken to confirm disc levels and heights. Rat tails were then affixed between the eighth and tenth coccygeal vertebrae using an Ilizarov-type device with springs. Axial force was applied from the distal side to produce a compressive stress of 1.3 MPa. This stress corresponds closely to a transient disc loading force produced by lifting a moderate weight in the human lumbar spine and induces morphological and biochemical disc degeneration. Following surgery, rats were divided into three groups (*N* = 3/group) according to loading duration: D1 group (loaded for 1 day then unloaded for 55 days), D7 group (loaded for 7 days then unloaded for 49 days), and sham group (unloaded for 56 days). This rat tail temporary static model reproduces different stages of IVD degeneration (D1: mild IVD degeneration; D7: moderate IVD degeneration) [18]. All IVD tissues were immunostained with AdipoR1 or AdipoR2 as described above.

### Cell culture and treatments

Coccygeal IVDs were aseptically dissected from eight SD rats. The nucleus pulposus (NP) and annulus fibrosus (AF) were then isolated separately. NP and AF cells were pre-cultured for 7 days at 37 °C in 5% CO_2_ and 95% air in complete tissue culture media containing Dulbecco’s modified Eagle’s medium (DMEM; Sigma Aldrich, St. Louis, MO, USA) supplemented with 10% fetal bovine serum (FBS, Sigma- Aldrich), 25 μg/ml ascorbic acid, 100 U/ml penicillin, and 100 mg/ml streptomycin. After pre-culture, the NP and AF cells were seeded into 6-well plates for complete adhesion and grown to approximately 60–70% confluence. The NP and AF cells were then treated with recombinant adiponectin (0.1 or 1.0 μg/ml; BioVision, Milpitas, CA, USA) and/or interleukin-1 beta (IL-1β, 0.2 μg/ml; R&D Systems, Minneapolis, MN, USA) for 24 h. The wells were divided into four groups as follows: (1) control group without IL-1β or adiponectin (Ad); (2) IL-1β group treated with IL-1β (0.2 μg/ml) only; (3) IL-1β+Ad (0.1) group treated with both IL-1β (0.2 μg/ml) and adiponectin (0.1 μg/ml); and (4) IL-1β+Ad (1.0) group treated with both IL-1β (0.2 μg/ml) and adiponectin (1.0 μg/ml).

### RNA extraction, reverse transcription, and quantitative RT-PCR analysis

Cultured NP and AF cells were collected and total RNAs were isolated using the RNeasy Mini Kit (Qiagen, Hilden, Germany) according to the manufacturer’s instructions. RNAs (0.1 μg) were reverse-transcribed in the presence of oligo d(T) primer (Applied Biosystems, Foster City, CA, USA).

Relative messenger-RNA (mRNA) expression levels of TNF-α and interleukin-6 (IL-6) were calculated by real-time reverse transcription-PCR using the ABI Prism7500 sequence detection system (Applied Biosystems). Glyceraldehyde 3-phosphate dehydrogenase (GAPDH) mRNA expression was measured as an endogenous control [18]. SYBR green fluorescent dye and predesigned primers (all obtained from Takarabio, Tokyo, Japan) were used according to the manufacturer’s instructions. The primer sequences used for PCR were as follows: GAPDH, sense 5′-GGCACAGTCAAGGCTGAGAATG-3′ and antisense 5′-ATGGTGGTGAAGACGCCAGTA-3′; TNF-α, sense 5′-TCAGTTCCATGGCCCAGAC-3′ and antisense 5′-GTTGTCTTTGAGATCCATGCCATT-3′; and IL-6, sense 5′-ATTGTATGAACAGCGATGATGCAC-3′ and antisense 5′-CCAGGTAGAAACGGAACTCCAGA-3′. The measurements were performed in duplicate, and mRNA expression levels of each gene in each experimental group were converted to a relative number representing the amount of mRNA compared with the control group using the 2^−ΔΔCt^ method [19].

### Statistical analysis

All values are expressed as means ± standard deviation (SD). Analysis of variance (ANOVA) with Fisher’s least significant difference (LSD) post hoc test was used to assess changes within and between experimental groups. Statistical significance was considered at a *P* value <0.05. Statistical analyses were performed using PASW Statistics 18 (IBM SPSS, Armonk, NY, USA).

## Results

### Adiponectin expression in human adipose tissues and rat IVD tissues

In human adipose tissues, immunohistochemical staining demonstrated expression of adiponectin in both human subcutaneous and epidural adipose cells. The mean percentage of positive adiponectin cells was 72.8 ± 14.9% in the subcutaneous and 67.8 ± 19.5% in the epidural adipose. No significant difference in adiponectin expression was identified between the subcutaneous and epidural adipose cells.

**Fig. 1:**
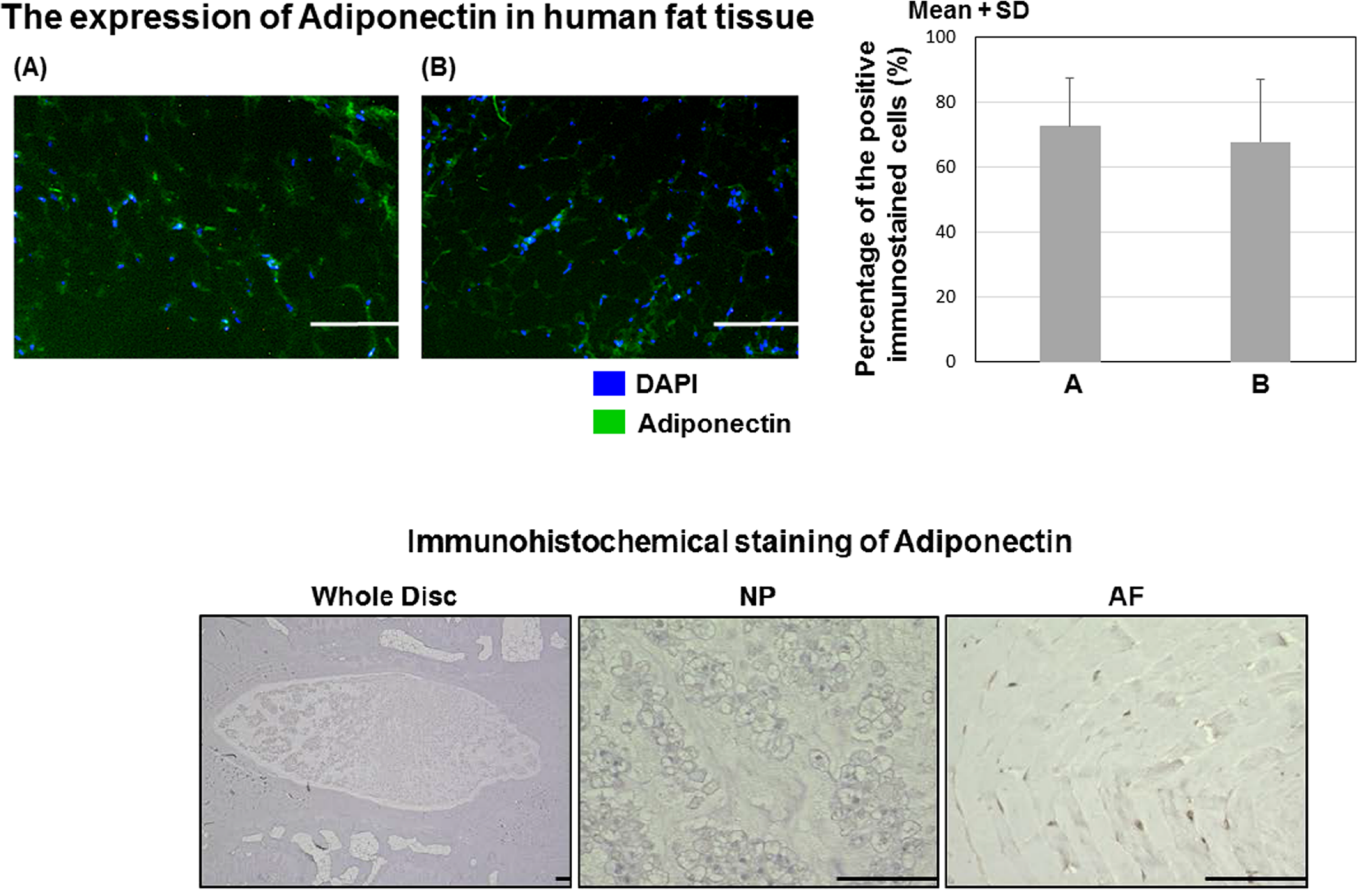
Immunohistochemical staining of adiponectin in human subcutaneous and epidural adipose tissue (*top left*). Photomicrographs demonstrating immunohistochemical localization of adiponectin in subcutaneous adipose tissue (*left*, *A*) and epidural adipose tissue (*left*, *B*); *bars* = 100 μm. Percentage (%) of positive immunostained cells (*top right*). Immunohistochemical staining of adiponectin in rat IVD tissue (*bottom*): no obvious staining was observed in either the NP or AF cells; *bars* = 100 μm

Conversely, in rat IVD tissues, adiponectin was not observed in both NP and AF tissues (Fig. 1).

### Adiponectin receptor expression in human and rat IVD cells

**Fig. 2:**
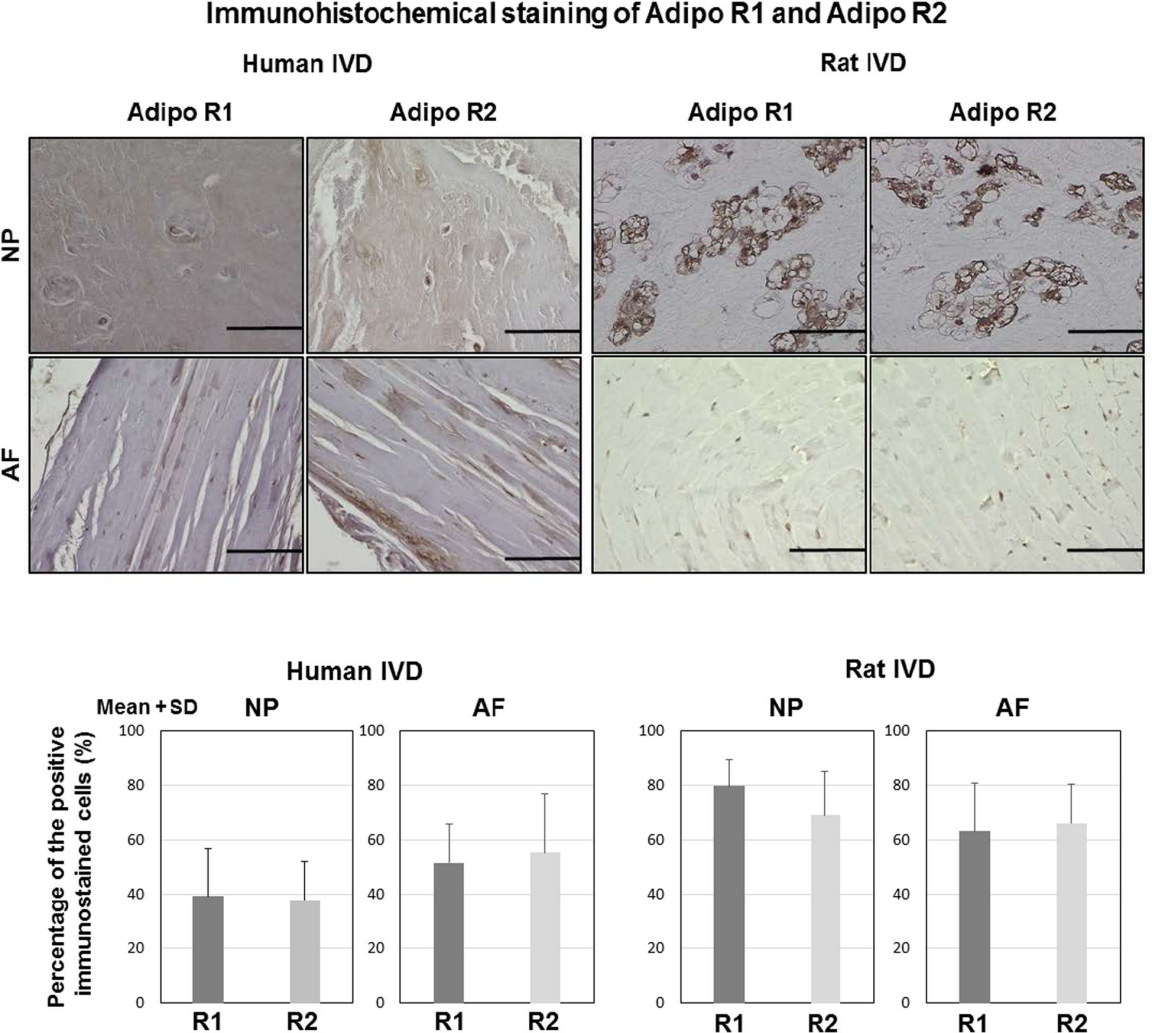
Immunohistochemical staining of AdipoR1 and AdipoR2 in human and rat IVDs. (*Top*) Photomicrographs demonstrating immunohistochemical localization of AdipoR1 and AdipoR2 in both NP and AF: *bars* = 100 μm. (*Bottom*) Percentage (%) of positive immunostained cells. Data were obtained from human IVD (*N* = 4) and rat IVD (*N* = 4), and expression is shown as mean + SD. *R1* AdipoR1, *R2* AdipoR2

AdipoR1 and AdipoR2 expression was observed in both NP and AF cells from human IVDs. The mean percentage of AdipoR1 and AdipoR2 was 39.4 ± 17.4% and 37.8 ± 14.5% in NP cells and 51.6 ± 14.3% and 55.4 ± 21.6% in AF cells, respectively. When expression levels of AdipoR1 and AdipoR2 were compared, no significant differences were observed for either NP or AF cells. Expression of AdipoR1 and AdipoR2 was observed in rat IVD cells: with a mean percentage of AdipoR1 and AdipoR2 at 79.6 ± 9.8% and 69.2 ± 15.8% in NP cells and 63.2 ± 17.5% and 66.2 ± 14.1% in AF cells, respectively. Expression of AdipoR1 and AdipoR2 was diffuse throughout the whole NP and AF. Localization of these receptors was not observed in human or rat IVD (Fig. 2).

### Identifying adiponectin receptor expression changes using a rat tail temporary static compression model

**Fig. 3:**
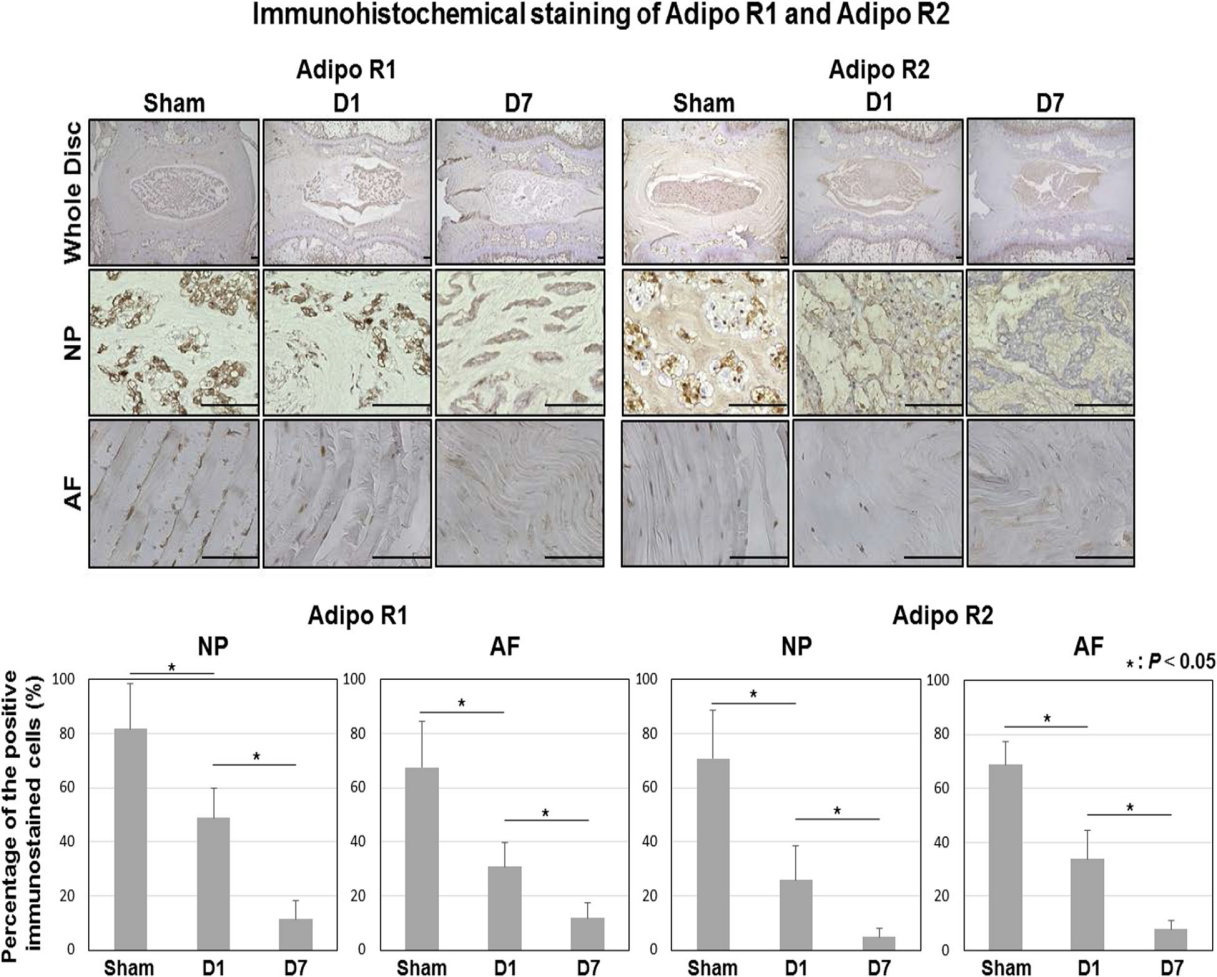
Immunohistochemical staining of AdipoR1 and AdipoR2 according to disc degeneration using a rat tail temporary static compression model. (*Top*) Photomicrographs demonstrating immunohistochemical localization of AdipoR1 and AdipoR2: *bars* = 100 μm in whole disc and NP and AF photographs. (*Bottom*) Percentage (%) of positive immunostained cells. Data were obtained from *N* = 9 and expression is shown as mean + SD

All animals tolerated surgery well and gained body weight throughout the experiment. All springs maintained their compressive length and were fully recovered immediately after release, indicating sustained axial loading and no apparent buckling. Infection, skin necrosis, neurological problems, or instrument failure were not observed. As shown in Fig. 3, AdipoR1 and AdipoR2 were widely observed in the sham group in both the NP and AF. Expression levels of AdipoR1 and AdipoR2 in both the NP and AF were gradually decreased with increased disc degeneration. AdipoR1 (% cells) decreased in the NP from 81.8 ± 16.7% (sham group) to 48.8 ± 10.8% (D1 group) and 11.4 ± 7.0% (D7 group) (*P* < 0.05) and in the AF from 67.6 ± 16.6% (sham group) to 30.8 ± 9.0% (D1 group) and 11.8 ± 5.5% (D7 group) (*P* < 0.05). AdipoR2 (% cells) decreased in the NP from 70.8 ± 17.6% (sham group) to 25.8 ± 12.6% (D1 group) and 4.8 ± 3.4% (D7 group) and in the AF from 68.8 ± 8.3% (sham group) to 33.8 ± 10.4% (D1 group) and 7.8 ± 3.3% (D7 group) (*P* < 0.05). In summary, these results showed that expression of both adiponectin receptors decreased in the NP and AF in accordance to the severity of IVD degeneration.

### TNF-α and IL-6 mRNA expression in rat IVD cells

**Fig. 4:**
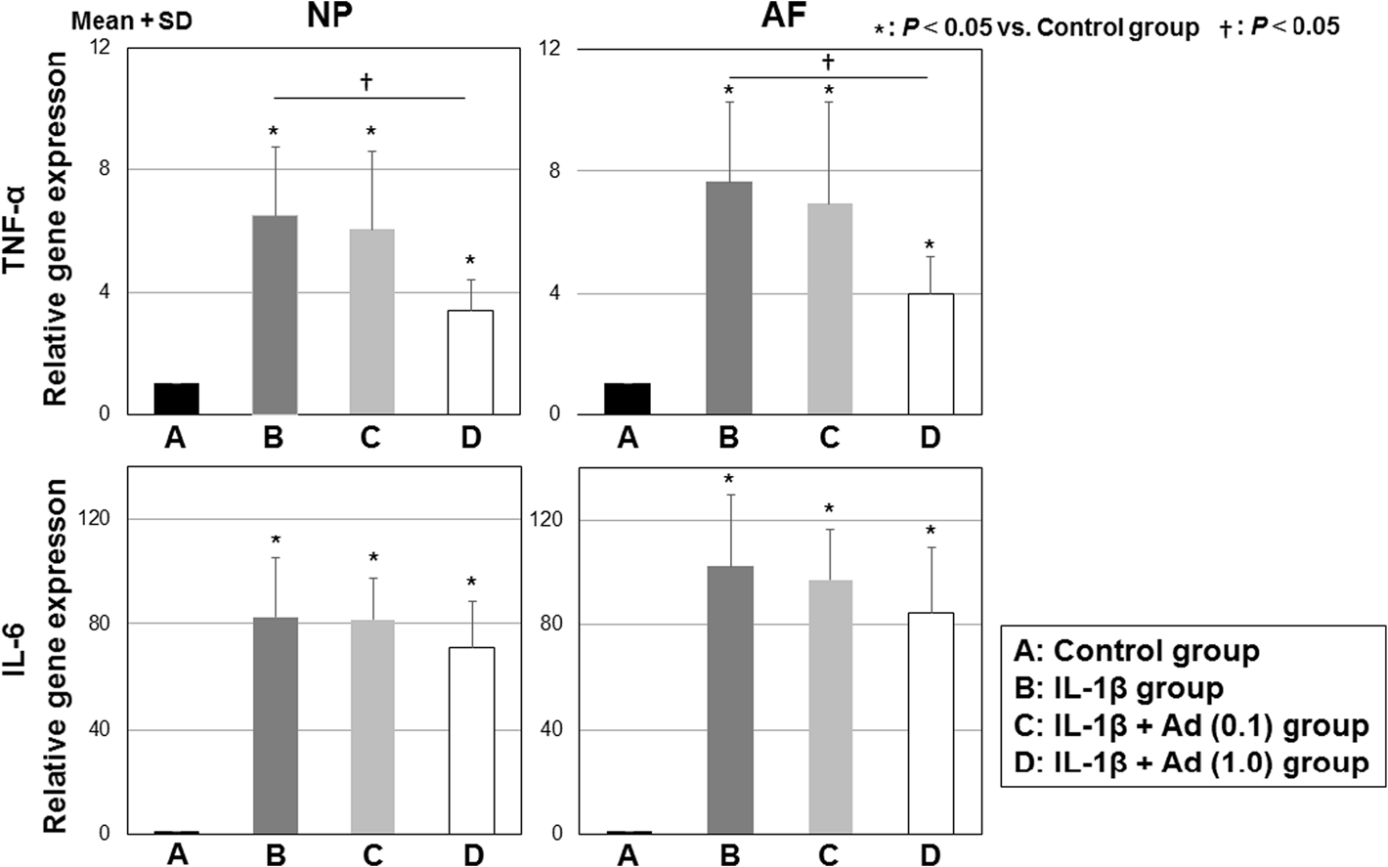
mRNA expression of TNF-α and IL-6 in rat NP and AF cells. *A*: control group without IL-1β and adiponectin; *B*: IL-1β group treated with IL-1β (0.2 μg/ml) only; *C*: IL-1β+Ad (0.1) group treated with both IL-1β (0.2 μg/ml) and adiponectin (0.1 μg/ml); and *D*: IL-1β+Ad (1.0) group treated with both IL-1β (0.2 μg/ml) and adiponectin (1.0 μg/ml)

As shown in Fig. 4, mRNA expression levels of pro-inflammatory cytokines, TNF-α and IL-6, were significantly upregulated by IL-1β treatment in both NP cells (+6.5-fold and +82.5-fold vs control, respectively; *P* < 0.05) and AF cells (+7.6-fold and +102.1-fold vs control, respectively; *P* < 0.05). The mRNA expression level of TNF-α in the IL-1β+Ad 1.0 group was significantly lower than that in the IL-1β group in both NP (−48.0%) and AF(−46.4%) cells (*P* < 0.05). Although mRNA expression of TNF-α in the NP and AF cells showed an identical trend in the IL-1β+Ad 0.1 group compared with the IL-1β group, a statistically significant difference was not reached. Interestingly, mRNA expression of IL-6 was not affected by adiponectin treatment in both the NP and AF cells. In summary, adiponectin inhibited IL-1β-induced expression of TNF-α in both the NP and AF IVD cells.

## Discussion

This is the first study reporting expression of adiponectin receptors (AdipoR1 and AdipoR2) in both human and rat IVD cells. Using a rat tail temporary static compression model, AdipoR1 and AdipoR2 expression were found to decrease according to disc degeneration severity. After assessing the anti-inflammatory effects of adiponectin on rat IVD cells, TNF-α expression induced by IL-1β was significantly downregulated by adiponectin treatment in cultured rat IVD cells.

AdipoR1 is known to be ubiquitously expressed, with high expression in skeletal muscle, while AdipoR2 is most abundantly expressed in the liver. Although these receptors are known to activate the AMPK and PPAR-α pathways, respectively, their functions remain unclear. Following reports of AdipoR1 and AdipoR2 expression in articular cartilage, bone, and synovial tissue [17], the current study investigated expression of these receptors in both human and rat IVD cells by immunohistochemistry. AdipoR1 and AdipoR2 were expressed at comparable levels in IVD NP and AF cells in humans and rats.

With respect to adiponectin, expression was not observed in rat IVD tissues. Adiponectin is thought to be supplied by diffusion from systemic or epidural adipose tissue. Whereas Khabour et al. reported that plasma adiponectin has strong association with severity of IVD degeneration, plasma adiponectin level was elevated in patients with IVD degeneration [20]. The association between serum adiponectin and bone mineral density (BMD) was also reported [21, 22]. In a recent review, IVD degeneration is characterized by degradation of aggrecan and collagen type II, promoted by an increase in pro-inflammatory cytokines such as TNF, IL-1β, and IL-6 [23]. In the current study, we focused on the anti-inflammatory effect of adiponectin and hypothesized that adiponectin uses this property as a protective role in IVD degeneration. Our results showed that adiponectin downregulated TNF-α expression induced by IL-1β stimulation in cultured IVD cells. Shibata et al. also reported that adiponectin inhibited TNF-α production using cultured neonatal cardiac myocytes and fibroblasts stimulated with lipopolysaccharide (LPS) [24]. They concluded that adiponectin suppresses LPS-induced secretion of TNF-α through a COX-2–prostaglandin E_2_–prostaglandin E_2_ receptor subtype 4 (EP_4_)-dependent pathway that is independent of AMPK signaling. Taken together, these and our current findings indicate that adiponectin elicits its anti-inflammatory effect by suppressing pro-inflammatory cytokines, especially TNF-α, and thus may have the potential to resist the IVD degeneration process due to pro-inflammatory cytokines.

We have previously reported the involvement of various factors, such as pro-inflammatory cytokines SIRT1, MMP-3, and kinematic mechanostress in IVD degeneration [25–30]. In 2014, Risbud and Shapiro reported that pro-inflammatory cytokines, including TNF-α and IL-1β, result in an imbalance in catabolic and anabolic responses leading to IVD degeneration and promote discogenic and radicular pain from the cytokine-mediated degenerative cascade [23]. In the current study, we used a rat tail temporary static compression model, representing the different stages of IVD degeneration, to investigate the involvement of adiponectin in IVD degeneration [18]. In the NP and AF, AdipoR1 and AdipoR2 expression gradually decreased with increased severity of disc degeneration. Based on the current study results, we consider that the anti-inflammatory function of adiponectin on IVD cells gradually weakens with the progression of IVD degeneration. In short, our findings suggest that adiponectin may be involved in the pathomechanism of IVD degeneration.

DM is a systemic disease that causes degenerative changes in many organs. Links between DM and IVDs, or spinal structure degeneration, have been reported using animal models. Fields et al. reported that glycosaminoglycan (GAG) and water content in the NP were significantly decreased in diabetic rats [6]. Also, diabetic rats showed that elevated expression of hypoxia-inducible genes and catabolic markers in the IVDs coincided with increased oxidative stress. Illien-Junger et al. reported that IVD height was significantly decreased in diabetic compared with non-diabetic mice [7]. DM is strongly associated with low plasma adiponectin levels and high incidence of LBP [12]; therefore, a biological approach using adiponectin may represent a suitable treatment option.

Several limitations of this study exist. The anti-inflammatory effect of adiponectin on rat and human degenerative IVD cells was not investigated. In addition, the function of adiponectin on the homeostasis of IVD cells needs to be determined in vivo. Finally, a suitable DM animal model should be used to determine the involvement of adiponectin in LBP of DM patients.

In conclusion, we have demonstrated expression of adiponectin receptors in the NP and AF tissues of human and rat IVD, the levels of which were determined by IVD degeneration severity. Adiponectin inhibited the TNF-α expression induced by IL-1β stimulation in rat NP and AF cells. Because adiponectin was not observed in the NP and AF cells, adiponectin is most likely supplied from the systemic or epidural adipose tissues and may be involved in IVD cell homeostasis.

## Conclusions

The expression of adiponectin receptors in the NP and AF tissues of human and rat IVD was observed. The expression levels were determined by IVD degeneration severity. Adiponectin inhibited TNF-α expression induced by IL-1β stimulation in rat NP and AF cells. Adiponectin was not observed in NP and AF cells; adiponectin is most likely supplied from the systemic or epidural adipose tissues. Adiponectin may have a potential to be involved in IVD cell homeostasis.

## Abbreviations

AdipoR1: Adiponectin receptor type 1
AdipoR2: Adiponectin receptor type 2
AF: Annulus fibrosus
AMPK: Activated protein kinase
BMD: Bone mineral density
DM: Diabetes mellitus
EP_4_: Prostaglandin E_2_ receptor subtype 4
GAG: Glycosaminoglycan
IL-1β: Interleukin-1 beta
IL-6: Interleukin-6
IVD: Intervertebral disc
LBP: Low back pain
LPS: Lipopolysaccharide
NP: Nucleus pulposus
PPAR-α: Peroxisome proliferator-activated receptor alpha
TNF-α: Tumor necrosis factor-α
